# Impact of Biocompatible Nanosilica on Green Stabilization of Subgrade Soil

**DOI:** 10.1038/s41598-019-51663-2

**Published:** 2019-10-22

**Authors:** Foad Buazar

**Affiliations:** Department of Marine Chemistry, Khorramshahr University of Marine Science and Technology, P.O. Box 669, Khorramshahr, Iran

**Keywords:** Geochemistry, Biosynthesis

## Abstract

This study reports the synthesis and potential application of biocompatible silica nanoparticles for subgrade soil stabilization. Nanosilica preparation as a major component from wheat husk ash is systematically studied and confirmed by FTIR, ICP, XRD, and TEM analyses. The produced biogenic nanosilica showed an amorphous structure with an average size of 20 nm. Upon loading various green nanosilica contents, our results show an improvement in the key parameters including Atterberg’s limits, maximum dry density, optimum water content, and shear strength of treated soil. Under optimal loading condition, the nanosilica-mediated soil analyses reveal a significant increase in the plastic and liquid limits by factors of 1.60 and 1.24 whereas plasticity index is declined by a factor of 0.78 rather than untreated soil specimen. The treated soil demonstrates a superior increase in the angle of internal friction, cohesion, shear strength, and maximum dry unit weight by factors of 2.17, 3.07, 2.21 and 1.5, respectively. The California Bearing Ratio (CBR) strength of nanosilica-cured soil presents a substantial increase by a factor of 5.83 higher than the corresponding original subgrade soil. We obtained the maximum increase in strength parameters of modified soil at the optimum biogenic nanosilica content of 1.5%.

## Introduction

The common inferior bearing capacity of weak subgrade soils has become a costly issue for the railroad foundation. They regularly contain high moisture, significant settlement, less bearing capacity, and low shear strength. The main objective of loose sub-grade soil treatment is the reduction in settlement and improving the load-bearing capacity to support various foundations purposes^1^. Hence, over the decades, stabilization has been one of the desired methods to enhance the efficiency of subgrade soils strength properties^2^. It is the changes in the soil to increase its physical properties to meet an engineering purpose and could be used on pavements, roadways, airports, railway, and several other locations when subsoils are unsuitable for construction^3^. Moreover, this method is able to treat a broad spectrum of sub-grade materials ranging from expansive soils to granular materials. Stabilization process can be divided into three different types including chemical, physical and mechanical methods. In mechanical approach, stabilization is obtained without changing the composition of the soil, namely, by dewatering and compaction while chemical stabilization is accomplished by loading different types of materials to change the nature of weak soils. Typically, chemical stabilizers comprise fiber^4^, fly-ash^5^, lime^6^, agricultural waste materials^7^, bitumen^8^ and cement^9^ to enhance weak soil tensile strength^10^. Specifically, fly ash, cement and lime are leading inorganic additives used in the soil stabilization. Due to their calcium-based structure, they frequently endure chemical reactions with the soil in the presence of water resulting in an overall improvement of the soil physicochemical properties^11^. Among these additives, fly ash owing to cost-effectiveness and abundance, dominantly attracts more consumers rather than lime in the construction industry.

Nonetheless, conventional soil stabilizers have a number of inherent disadvantages, such as high cost of excessive maintenance, poor soil structure, secondary chemical pollution and detrimental environmental impacts associated with construction^12^. Therefore, nanoparticles additives due to their unique characteristics are proposed as an emergent alternative stabilizer to overcome at least some of the detriments of traditional soil stabilization approaches^13,14^. Previous studies have demonstrated that nanoscale particles lead to tangible changes to mechanical, physical, and chemical properties of treated soil^15–18^. For instance, Fe_2_O_3_ nanoparticles is employed as filling agents to bundle the pores and strengthen concrete. Their combination with fly ash as a cement substitution can also strengthen the mechanical characteristics of concrete^19^. Furthermore, inorganic nano-additives such as Al_2_O_3_, CuO, and clay nanopowder effectively improved the engineering properties of the weak soils^20^. Zhang *et al*. indicated that increasing chemical SiO_2_ nanoparticles improves unconfined compressive strength and triggers denser packing as well as stiffness behavior of cured loess^11^. Moreover, it is found that Atterberg’s limits values of clays increase upon addition of nanoclay materials^21^.

**Figure 1:**
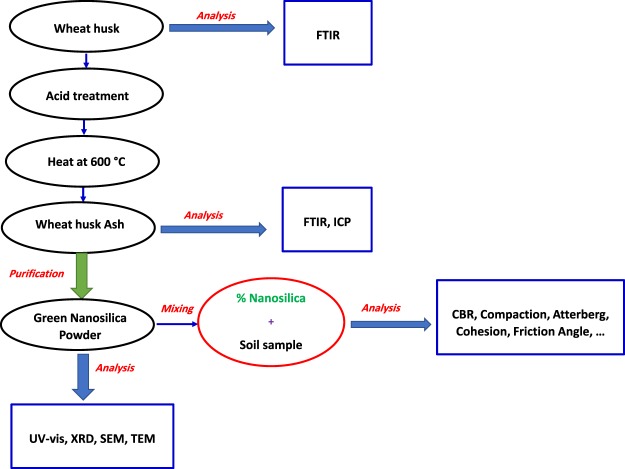
Schematic illustration of green nanosilica production for subgrade soil stabilization.

Silica nanostabilizer hold promising prospective material for weak soil stabilization on account of cost-effectiveness, high durability characteristics, and reliable stabilization^22–24^. In this connection, few reports have investigated the influence of silica nanoparticles (NPs) on the geotechnical properties of different form of stabilized soils^21,24,25^. To the best of our knowledge, no study has found in the literature concerning the effect of plant-mediated nanosilica additive on subgrade soil for railway construction purpose. Consequently, following our interest in green synthesis and application of nanomaterial^26–28^, the underlying goal of this study is to evaluate the main mechanical properties of weak subgrade soil in combination with wheat husk-assisted produced silica NPs as a novel biocompatible nanostabilizer (Fig. 1). The wheat husk is considered as wheat lignocellulosic waste material which is easily available in 15–20% of wheat and normally employs for livestock nutrition^29^. It exposes a high potential for application in the synthesis of green silica NPs owing to its appropriate ratio of silicate content.

## Result and Discussion

### Properties of wheat husk

**Figure 2:**
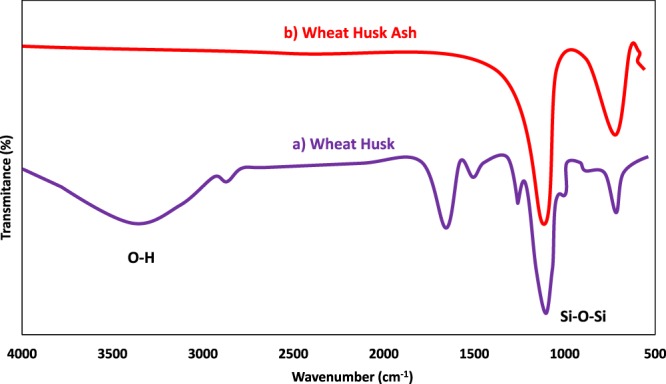
(**a**) FTIR spectra of raw wheat husk and (**b**) wheat husk ash.

FTIR spectrum of wheat husk is depicted in Fig. 2a. The characteristic absorption peaks of prominent functional groups appear in 3320, 2915, 1738, 1412, 1130, 1052, 1012, and 785 cm^−1^ bands, respectively. The visible broad peak at 3320 cm^−1^ can be ascribed to the O–H bond stretching vibration of adsorbed water and the band at 2915 cm^−1^ also indicated *v*(CH_2_) stretching vibration. The peak at 1738 cm^−1^ can be attributed to C=O stretching of polysaccharides such as hemicellulose and/or lignin present in the wheat husk. The symmetric and asymmetric stretching vibration of ethereal C-O-C groups occurr at 1130 and 1012 cm^−1^ can be assigned to ester linkage between the sugar units. In addition, the distinctive signal at around 1052 cm^−1^ presents siloxane (O-Si-O) functional group. The observed band at 785 cm^−1^ is related to the stretching vibration of the silanol (Si–OH) group. However, upon heating wheat husk at 600 °C for 6 h in the furnace, all the bands of organic matters of fresh wheat husk disappear and hence the FTIR spectrum of its ash only exhibits sharp peak at 1065 cm^−1^ and the minor peak at 792 cm^−1^ representing the Si-O-Si stretching vibrations (Fig. 2b). Likewise, Yucel and Terzioglu demonstrated that absorbance frequency of SiO_2_ extracted from home-grown wheat husk ash reveals at 1032 cm^−1^ band^30^.

### Chemical composition

The chemical contents of wheat husk ash obtained at 600 °C are illustrated in Table 1. The ash is composed of SiO_2_, K_2_O, CaO, MgO, Na_2_O, Fe_2_O_3_, MnO_2_, Cr_2_O_3_, and Al_2_O_3_. Silicon dioxide (SiO_2_) is the significant constituent of the ash with 48.12%. Other prominent components are detected as potassium oxide (K_2_O, 10.97%) and calcium oxide (CaO, 5.15%). The remaining chemical composition can be anticipated to the residual elements of ash and other unburnt organic substances. Similarly, previous reports demonstrated that the highest proportion of raw rice husk is belonged to SiO_2_ component thus confirming agricultural waste materials as a sustainable source for the production of green silica NPs^31^.

**Table 1:** Chemical compositions of domestic wheat husk ash.

Compound	SiO_2_	K_2_O	MgO	Fe_2_O_3_	Na_2_O	Cr_2_O_3_	MnO_2_	CaO	Al_2_O_3_
wt%	48.12	10.97	1.09	0.88	0.21	0.0003	0.03	5.15	0.13

#### Properties of nanosilica stabilizer

**Figure 3:**
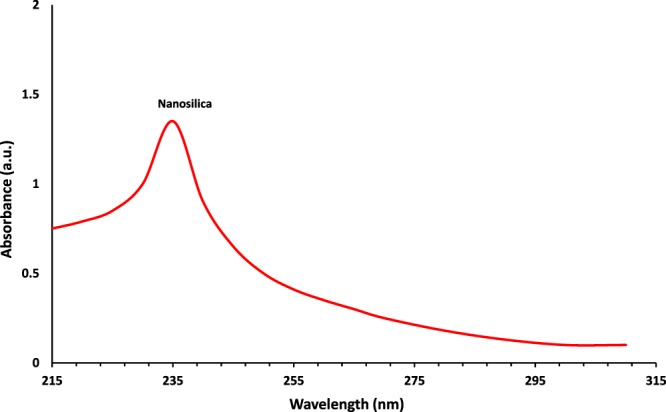
UV-vis absorption spectrum of biogenic silica NPs synthesized using wheat husk ash.

**Figure 4:**
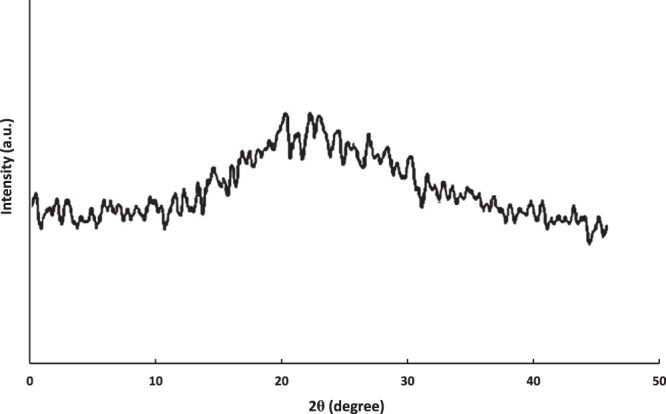
XRD pattern of amorphous green silica NPs fabricated using wheat husk ash.

Figure 3 shows the UV-vis spectrum of as-prepared green silica NPs. Initial visual observation after burning the silica precursor (wheat husk ash) and presence of an absorption peak at 235 nm indicated the successful formation of white-colored silica NPs from the fresh wheat husk. According to XRD pattern results, a fairly wide band at 2θ = 23° of Bragg angle confirmed that the crystal structure of green silica nanopowder is amorphous in nature (Fig. 4)^32^. Owing to the amorphous crystalline structure, the biosynthesized silica NPs, could situate properly in the bedrock layers of raw soil^33^.

**Figure 5:**
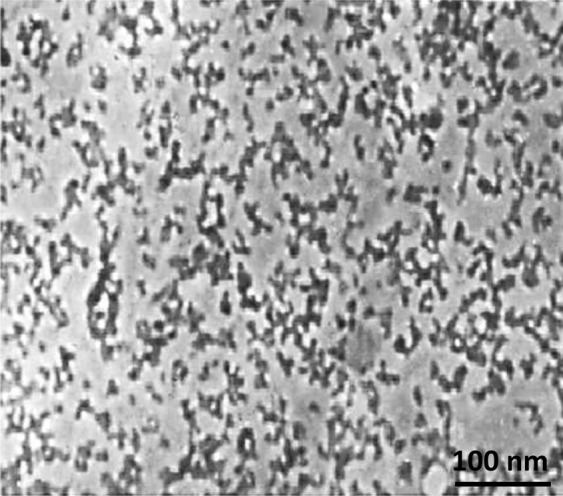
TEM image of biosynthesized silica NPs using wheat husk ash.

The surface morphology of the bio-assisted nanosilica is depicted in Fig. 5. Analysis of the TEM image reveals that bioprepared silica nanomaterial has an unstructured shape which supports XRD results (see Fig. 4). These results are consistent with other reports in which rice husk and wheat hull-mediated silica NPs are predominantly amorphous in shape^34–36^. The average particle size of the majority of nanosilica particles is approximately around 20 nm. Taking into account the high surface energy, biological silica NPs displays arbitrary aggregations, thus leading to formation of large particle clusters with poor dispersivity.

#### Properties of raw subgrade soil

The main mechanical and physical properties of collected natural subgrade soil sample are presented in Table 2. The raw subgrade soil used in the investigation is classified as low plasticity clay (CL) according to Unified Soil Classification System^37^. The color of the crude subgrade soil is mainly light brown and relatively associated with a high proportion of clay. According to Atterberg’s limits results, the parent subgrade soil exposes poor mechanical features and as a result, it is ranked in weak soils category.

**Table 2:** Properties of raw subgrade soil.

Properties	Result
Maximum dry density (kN/m^3^)	1.81
Sand (%)	19
Fine-grained (%)	81
USCS classification	CL
Liquid limit (%)	38
Plastic limit (%)	20
Shrinkage limit (%)	17
Optimum moisture content (%)	19.4
Cohesive strength	15
Angle of internal friction	27
CBR	12

#### Effect of green nanosilica on the optimum moisture content and maximum dry unit weight of subgrade soil

**Figure 6:**
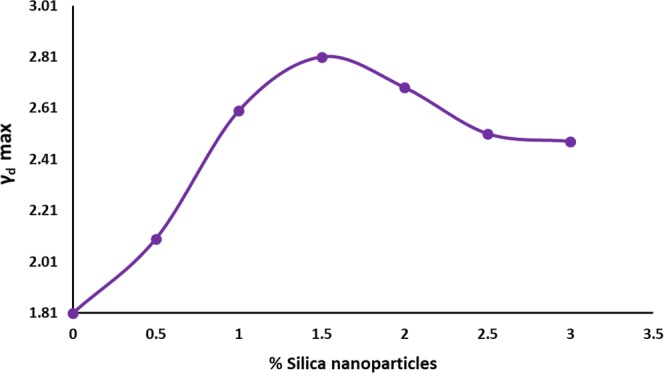
Effect of green silica NPs on the maximum dry density of subgrade soil.

**Figure 7:**
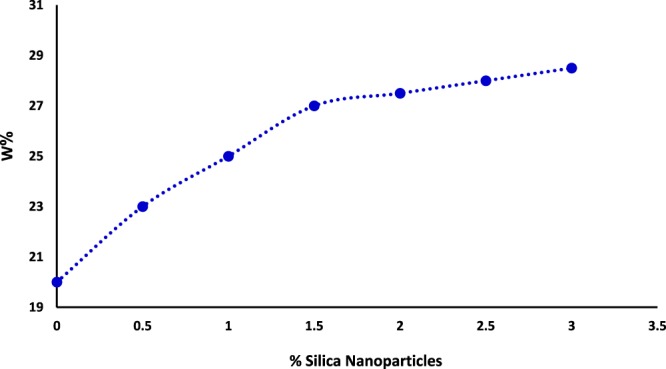
Effect of green silica NPs on the optimum moisture content of subgrade soil.

Figure 6 shows the results of the maximum dry unit weight and the optimum moisture content of nanosilica-stabilized soil using compaction test (ASTM D698-78)^38^. Results show that increase in the percentage in green nanosilica content leads to steadily boost in the maximum dry unit weight (γdmax**)** of nanosilica-stabilized soil. As shown in Fig. 4, the addition of nanosilica stabilizer up to 1.5% provides the highest amount of the dry density for the subgrade soil which is probably due to the appropriate replacement of nanosilica additive with air in soil pores^39^. An increase in the weight of silica NPs over an optimal of 1.5% leads to a decrease in the volumetric density of soil as a result of the accumulation and agglomeration of silica NPs. Virtually, the optimum moisture content of silica-treated subgrade soil is effectively improved by embedding different proportions of nanosilica (0.5, 1, 1.5, and 2%) (Fig. 7). The incorporated silica NPs in the soil are apparently absorbed by intergranular water, which in turn cause the pores in the soil be filled with nanosilica. Moreover, due to high ion charge, silica–stabilized soil most likely absorbs hydrated ions and thus, enhances soil moisture content^21^.

#### Effect of green nanosilica on the Atterberg’s limits of subgrade soil

**Figure 8:**
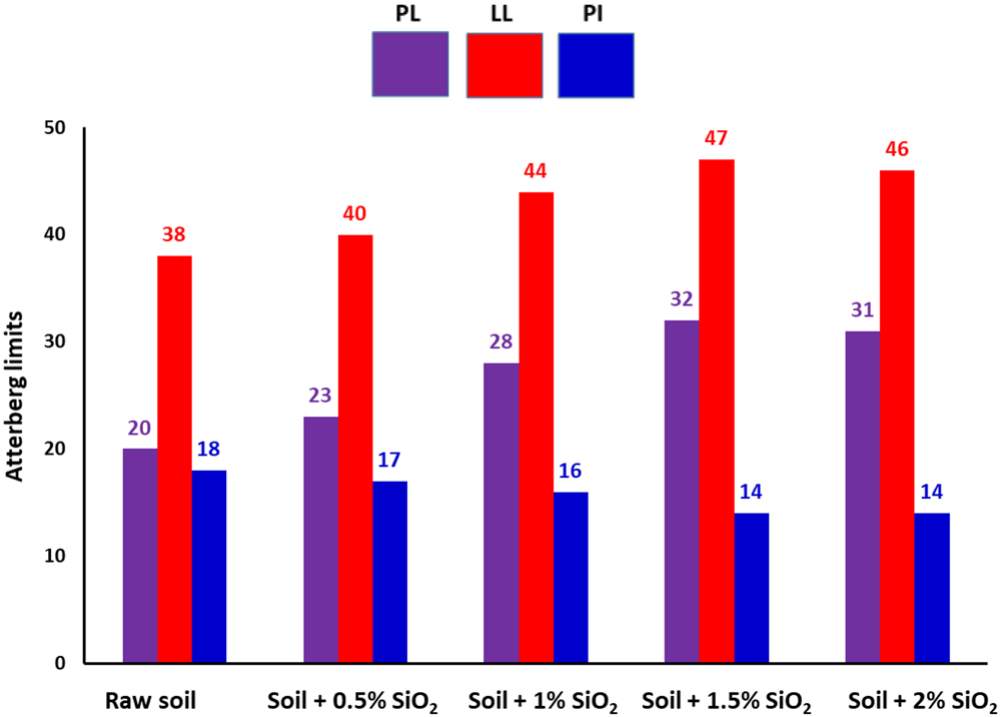
The effects of green silica NPs on the plastic limit (PL), liquid limit (LL), and plasticity index (PI) of raw subgrade soil.

Atterberg’s limits parameters are a pivotal measure of the critical water contents of a fine-textured soil^40^. Figure 8 displays the result of plastic limit (PL), liquid limit (LL), and plasticity index (PI) of nanosilica-stabilized soil and natural subgrade soil as well. According to values of LL = 38, PL = 20, and PI = 18, the studied subgrade soil is classified as clay soil of low plasticity (CL). Interestingly, by adding different amount of silica nanostabilizer into the soil composition the values of Atterberg’s limits steadily enhance higher than corresponding pristine subgrade soil. Yet, at the optimal nanosilica content of 1.5%, the amount of LL, PL, significantly increase by factors of 1.29 and 1.6, respectively. Apparently, silica NPs could efficiently react with water molecules in soil texture due to chemical nature and high ratio of the surface to the volume. Consequently, water is accumulated in silica nanopores which in turn increase the water capacity in the subgrade soil. Similarly, earlier reports indicated that addition nanoclay to the clay effectively increases the liquid and plastic limit as well^41^. In addition, PI criteria of raw subgrade soil decreased proportionally with constant nanosilica addition, indicating the reduction of its plastic properties^42^. The shrinkage limit (SL) amount of 17 was determined as the maximum value to saturate subgrade soil pores spaces.

#### Effect of green nanosilica on the cohesion and the angle of internal friction of subgrade soil

**Figure 9:**
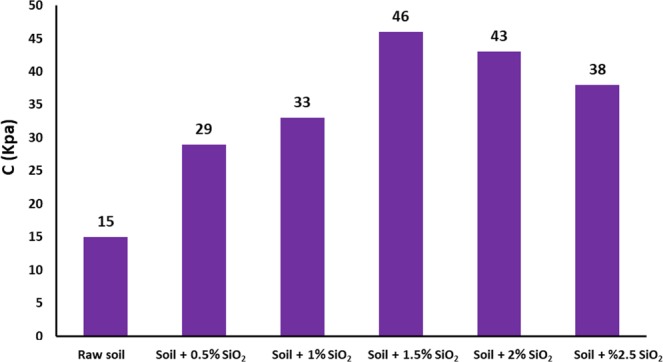
The effect of varying biological silica NPs content on subgrade soil cohesion (C).

The cohesion results of raw and nanosilica-mediated subgrade soil is illustrated in Fig. 9 using direct shear test (ASTM D2216-90)^43^. Shear strength of the soil is crucial to determine the soil stability for bases construction. In comparison to untreated soil, incorporating different concentrations of silica nanostabilizer of 0.5, 1, 1.5, 2, and 2.5%, in soil texture reveals a significant increase in cohesion values by factors of 1.93, 2.20, 3.06, 2.87, and 2.53, respectively. The maximum strength of subgrade soil was achieved by 1.5% nanosilica additive. Besides, cohesion of stabilized soil substantially increased to 49 Kpa higher than corresponding basic subgrade soil (15 Kps). The results indicate that increment different proportions of green nanosilica often lead to an effective increase of soil cohesion and therefore, enhances the strength of nanosilica-stabilized soil^44^.

**Figure 10:**
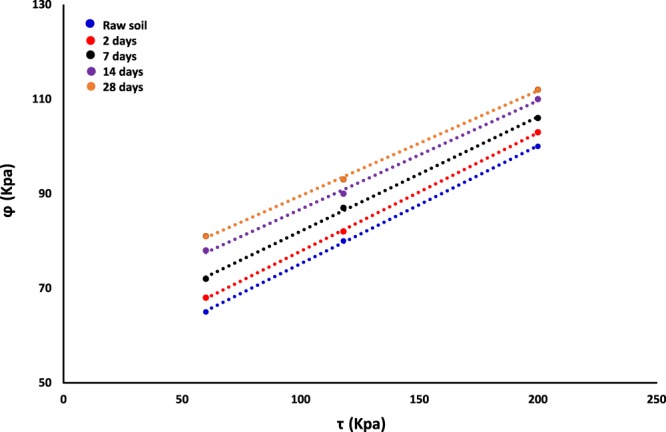
The effect of green nanosilica additive on internal friction angle (φ) of subgrade soil.

Figure 10 illustrates the changes of the angle of internal friction (φ) and shear strength of raw and nanosilica-stabilized soil performed at room temperature for 2, 7, 14 and 28 days under three stress values of 60, 118, and 200 Kpa, respectively. The results indicate that the addition an optimal amount up to 1.5% nanosilica results in the maximum shear strength and an increase in the angle of internal friction higher than untreated soil. Due to high viscosity, the presence of the nanosilica stabilizer in raw soil composition could function as robust cross-linker between internal soil particles, and hence, it improves the major criteria including cohesion, the angle of internal friction and the inner strength of nanosilica-cured subgrade soil.

#### CBR results

California bearing ratio (CBR) is a major criterion to measure the physical strength and weakness of natural and treated soil for the construction of subgrade railroad, pavement, subbase, and road as well^45^. Based on CBR standard test (AASHTO T193)^46^, using a cylindrical rod gadget of CBR experiment, mixing 1.5% green nanosilica content with the soil specimen demonstrates that the rate of CBR strength is approximately 5 times greater than that of corresponding parent subgrade soil (see Table 1), indicating significant improvement in bearing capacity of cured subgrade soil samples for railway track designing. In an earlier study, CBR test analysis exhibited that the soil sample containing nanoclay proportion of 1.5 wt% shows higher CBR value than raw soil, supporting our findings^47^.

## Conclusion

This study presents the results of successful biosynthesis of green silica nanoparticles from waste products of wheat plant. The characterization of as-prepared silica nanoparticles and fresh wheat husk was investigated using FTIR, ICP, UV-vis, XRD, and TEM techniques. The biofabricated silica nanoparticles exhibit an amorphous morphology with an average size of 20 nm. Further, the effect of green nanosilica additives (0.5, 1, 1.5, and 2% of the total dry weight) on subgrade soil stabilization was studied. Our results indicat that adding nanoscale silica particles efficiently increase key parameters including optimum moisture content, the maximum dry density, cohesion, and the angle of internal friction of natural subgrade soil. The highest increase in geotechnical properties of nanosilica-stabilized soil occurrs in an optimal percentage of nanosilica at 1.5%. Adding low content of ecofriendly nanosilica additive considerably enhances the CBR strength of weak subgrade soil. Finally, this innovative and green stabilization approach could induce further sustainability and biocompatibility for railroads infrastructure and also tackle hazardous materials employed in weak soil stabilization.

## Materials and Methods

### Chemical

Analytical grade nitric acid (HNO_3_, 90%), and sodium hydroxide (NaOH) were obtained from Aldrich and used without further purification. Deionized distilled water is used in the preparation of all solutions. Wheat husks were collected from Ahwaz province wheat farms, south of Iran.

### Subgrade soil samples

The loose subgrade soil samples with light grey color were collected from Ahwaz-Khorramshahr railroad, south of Iran (Fig. S1, see supplementary), using the zigzag sampling method^48^. All the soil samples were crashed by a hammer then screened through 4.75 mm size sieve to make them free from roots, pebbles, gravel, and other contaminants. Then they were sealed and wrapped with plastic after gathering to maintain the original moisture contents and stored at room temperature in the laboratory. The tests were carried out on loose subgrade soil samples for physical and engineering properties in accordance with international standard methods (Table 1). All nanosilica-stabilized soil samples were obtained from field test operated in the natural environment (Fig. S2, supplementary).

### Biogenic silica NPs synthesis

In a typical recipe, the contaminated raw wheat husks as silica precursor were washed with tap water and dried in the oven at 60 °C for 24 h to obtain pure material. The powdered wheat husk (15 g) was dispersed in 500 mL of 10% H_2_SO_4_ aqueous solution and heated at 90 °C for 3 h under vigorous stirring to remove the impurities. The solid residue is filtered and washed with deionized water to eliminate the remained sulfate salt. Finally, the resultant is calcined in a furnace at 600 °C for 2 h to achieve fine white silica nanopowder via removal of unburned carbon-derivative materials.

### Wheat husk characterization

The surface chemistry and absorption bands of functional groups of fresh wheat husk and its ash powder are examined by Fourier transform infrared spectroscopy (Brucker, VERTEX 70, Germany) in the range of 500 to 4000 cm^−1^. The chemical compositions of wheat husk ash are detected using an Inductively Coupled Plasma-Mass spectrometry (Agilent 7800 ICP-MS, Japan).

### Nanosilica characterization

The formation, crystal structure, morphology, and particle size of biosynthesized silica NPs are investigated using UV-Vis (Analytic Jena-Germany), XRD pattern (MPD from analytical), and TEM (Zeiss-EM10C-100KV-Germany) techniques, respectively.

### Nanosilica-soil mixture preparation

Four different percentages of green nanosilica content (0.5, 1, 1.5, and 2% by weight of the raw soil), were selected in this investigation (Table 1). In order to prepare specimens stabilized with green nanosilica, the quantity of natural subgrade soil is divided into four portions and each part is blended with the defined percentage of biogenic nanosilica additive. To obtain a homogenous mixture, the optimal amount of water is gradually added to nanosilica-treated soil samples and soaked for 1 h. For comparison, the standard soil testing methods including compaction and shear experiments were conducted on both modified and natural soil specimens. Moreover, the prepared mixture also is used to measure maximum dry unit weight and optimum water content as well (Fig. 1). All related measurements were repeated three times and acquired average values were reported.

## Supplementary information

Supplementary
