# Supplementary material for: Impact of Biocompatible Nanosilica on Green Stabilization of Subgrade Soil
Source: Sci Rep. 2019 Oct 22;9:15147. doi: 10.1038/s41598-019-51663-2 (PMC6805850; doi:10.1038/s41598-019-51663-2)
Supplement: Supplementary file 1 — Supplementary [file 41598_2019_51663_MOESM1_ESM.pdf]

Impact of Biocompatible Nanosilica on Green Stabilization of Subgrade Soil

Foad Buazar*

Department of Marine Chemistry, Khorramshahr University of Marine Science and Technology, P.O. Box 669, Khorramshahr, Iran

* Corresponding author, Tel: +98-916115084, Email: fb@kmsu.ac.ir

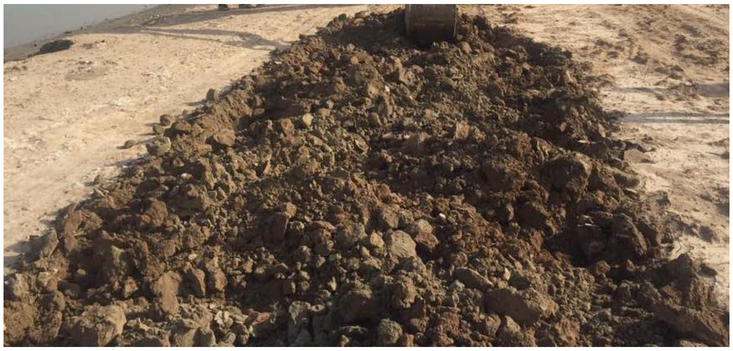

Figure S1. Illustration loose subgrade soil from Ahwaz-Khorramshahr road

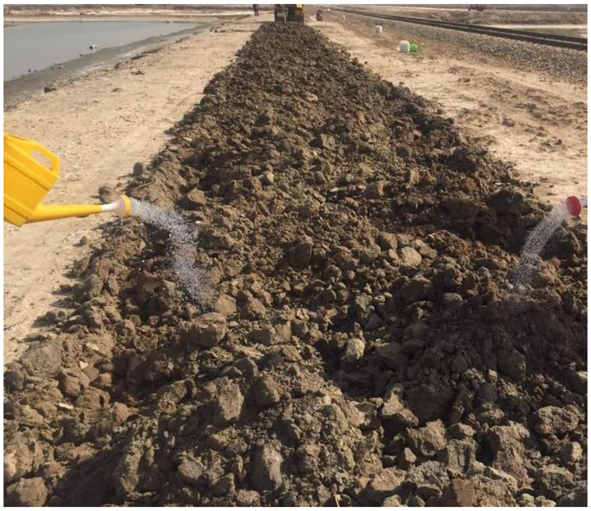

Figure S2. Experimental test of the parent soil treatment with green silica nanoadditives in Ahwaz-Khorramshahr road
